# Characterization of the complete mitochondrial genome of Xanthid crab, *Atergatis integerrimus* from China (Decapoda: Brachyura) and its phylogenetic analysis

**DOI:** 10.1080/23802359.2018.1456374

**Published:** 2018-03-27

**Authors:** Zhuofang Xie, Huaqiang Tan, Fan Lin, Mengyun Guan, Khor Waiho, Shaobin Fang, Mhd Ikhwanuddin, Hanafiah Fazhan, Hongyu Ma

**Affiliations:** a Guangdong Provincial Key Laboratory of Marine Biotechnology, Shantou University, Shantou, China;; b Institute of Tropical Aquaculture, Universiti Malaysia Terengganu, Kuala Terengganu, Terengganu, Malaysia

**Keywords:** Xanthid crab, *Atergatis integerrimus*, mitochondrial genome, phylogenetic analysis

## Abstract

The complete mitochondrial genome sequence of *Atergatis integerrimus* from China has been amplified and sequenced in this study. The mitogenome assembly was found to be 15,924 bp in length with base composition of A (32.88%), G (10.58%), C (20.87%), T (35.66%), A + T (68.54%), and G + C (31.46%). It contained 13 protein-coding genes, 22 transfer RNA genes, two ribosomal RNA genes and a control region. The phylogenetic position was constructed and the *A. integerrimus* was closely clustered with *Pseudocarcinus gigas* and *Leptodius sanguineus*. The complete mitochondrial genome sequence would be useful for further understanding the evolution of *A. integerrimus*.

*Atergatis integerrimus* belongs to the Family Xantidae in the order Decapoda. Also named as the Rubble crab or Red Egg crab, *A. integerrimus* is one of the most colourful large crab species often encountered in coral reef and rocky area around Indo-Pacific region (Gopalakrishnakone 1990; Davie 2002; Wisespongpand et al. 2013). Complete mitochondrial genome is getting attention as the new genome marker is used to differentiate the organism to the species level especially the crab’s taxa instead of using partial and shorter mitochondrial gene sequences (Ma et al. 2015). Until now, about 67 species mitochondrial genome of crabs were successfully sequenced and published in the gene bank. Out of the 67 species, only three species are from Superfamily Xanthoidea, i.e. two species from the family Xantidae (*A. integerrimus* (Philippines strain) and *Leptodius sanguineus*) and one from the family Mennipidae (*Pseudocarcinus. gigas*). Due to the limited number of crabs’ mitogenome from the family Xantidae and the usefulness of mitogenome in discerning species and in-depth phylogenetic and population studies, the complete sequence of mitochondrial genome of *A*. *integerrimus* from China is made available in this study.

Specimen of *A. integerrimus* was collected from, Weizhou Island (21.0234°N, 109.0940°E), Guangxi province, China and deposited in the Marine Biology Institute, Shantou University, Shantou, China. Total genomic DNA was isolated from the muscle tissue and subjected to long and conventional PCR to obtain the complete mitochondrial genome sequence. The maximum-likelihood (ML) phylogenetic tree based on 12 concatenated protein coding genes (PCGs) of mitochondrial genome was constructed together with nine complete mitogenomes (*Pseudocarcinus gigas* (NC006891), *Leptodius sanguineus* (NC029726), *Thalamita crenata* (LK391945), *Portunus pelagicus* (KT382858), *Geothelpusa dehaani* (NC007379), *Cyclograpsus granulosus* (NC0255710), *Scylla paramamosain* (JX457150), and *Sesarmop sinensis* (NC030196), with *Panulirus japonicus* (NC004251) served as an outgroup) retrieved from National Center for Biotechnology Information (NCBI). The ML tree analysis of *A. integerrimus* together with the selected species and outgroup was performed using MEGA7: Molecular Evolutionary Genetics Analysis version 7.0 for bigger datasets (Kumar et al. 2016; Lin et al. 2018) with suggested model, GTR (G + I) and replicated 100 times bootstraps. The phylogenetic tree was viewed using ETE 3 toolkit (Huerta-Cepas et al. 2016).

The complete mitochondrial genome of *A. integerrimus* is 15,924 bp in length with base composition of A (32.88%), G (10.58%), C (20.87%), T (35.66%), A + T (68.54%), and G + C (31.46%) which is shorter compared to the mitogenome of *A. integerrimus* (16,333 bp) from the Philippines (Karagozlu et al. 2018). The mitogenome sequence of *A. integerrimus* with the annotated genes was deposited in GenBank under the accession number of MG786939. It contained 13 protein-coding genes, 22 transfer RNA genes, two ribosomal RNA genes and a control region. The phylogenetic position was constructed and the *A. integerrimus* was closely clustered with *P. gigas* and *L. sanguineus* (Figure 1).

**Figure 1.**
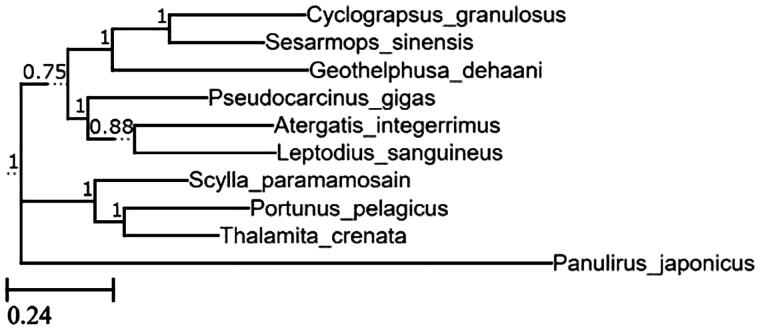
Phylogenetic relationships among concatenated mitochondrial 12 protein-coding genes without ND6 sequences of 10 mitochondrial genomes including *Panulirus japonicus* as the outgroup inferred using maximum-likelihood analysis.
